# Explainable Machine Learning in the Prediction of Depression

**DOI:** 10.3390/diagnostics15111412

**Published:** 2025-06-02

**Authors:** Christina Mimikou, Christos Kokkotis, Dimitrios Tsiptsios, Konstantinos Tsamakis, Stella Savvidou, Lillian Modig, Foteini Christidi, Antonia Kaltsatou, Triantafyllos Doskas, Christoph Mueller, Aspasia Serdari, Kostas Anagnostopoulos, Gregory Tripsianis

**Affiliations:** 1 Laboratory of Medical Statistics, School of Medicine, Democritus University of Thrace, 68100 Alexandroupolis, Greece; xmimikou@yahoo.com (C.M.); gtryps@med.duth.gr (G.T.); 2 Department of Physical Education and Sport Science, Democritus University of Thrace, 69100 Komotini, Greece; ckokkoti@affil.duth.gr; 3 3rd Department of Neurology, Aristotle University of Thessaloniki, 54124 Thessaloniki, Greece; savvids@gmail.com (S.S.); lilianmodig@gmail.com (L.M.); 4 Institute of Psychiatry, Psychology and Neuroscience (IoPPN), King’s College London, London SE5 8AB, UK; ktsamakis@gmail.com (K.T.); christoph.mueller@kcl.ac.uk (C.M.); 5 Department of Clinical Sciences, New Anglia University, George Hill AI-2640, Anguilla; 6 Department of Neurology, Democritus University of Thrace, 68100 Alexandroupolis, Greece; christidi.f.a@gmail.com; 7 Physical Education and Sport Science, University of Thessaly, 42132 Trikala, Greece; akaltsat@gmail.com; 8 Department of Neurology, Athens Naval Hospital, 11521 Athens, Greece; doskastr@gmail.com; 9 Department of Child and Adolescent Psychiatry, School of Medicine, Democritus University of Thrace, 68100 Alexandroupolis, Greece; aserntar@med.duth.gr; 10 Laboratory of Biochemistry, School of Medicine, Democritus University of Thrace, 68100 Alexandroupolis, Greece; kanagnos@med.duth.gr

**Keywords:** artificial intelligence, neural networks, logistic regression, support vector machine, XGBoost, interpretation

## Abstract

**Background:** Depression constitutes a major public health issue, being one of the leading causes of the burden of disease worldwide. The risk of depression is determined by both genetic and environmental factors. While genetic factors cannot be altered, the identification of potentially reversible environmental factors is crucial in order to try and limit the prevalence of depression. **Aim:** A cross-sectional, questionnaire-based study on a sample from the multicultural region of Thrace in northeast Greece was designed to assess the potential association of depression with several sociodemographic characteristics, lifestyle, and health status. The study employed four machine learning (ML) methods to assess depression: logistic regression (LR), support vector machine (SVM), XGBoost, and neural networks (NNs). These models were compared to identify the best-performing approach. Additionally, a genetic algorithm (GA) was utilized for feature selection and SHAP (SHapley Additive exPlanations) for interpreting the contributions of each employed feature. **Results:** The XGBoost classifier demonstrated the highest performance on the test dataset to predict depression with excellent accuracy (97.83%), with NNs a close second (accuracy, 97.02%). The XGBoost classifier utilized the 15 most significant risk factors identified by the GA algorithm. Additionally, the SHAP analysis revealed that anxiety, education level, alcohol consumption, and body mass index were the most influential predictors of depression. **Conclusions:** These findings provide valuable insights for the development of personalized public health interventions and clinical strategies, ultimately promoting improved mental well-being for individuals. Future research should expand datasets to enhance model accuracy, enabling early detection and personalized mental healthcare systems for better intervention.

## 1. Introduction

Depression, a chronic mood disorder characterized by loss of interest and a persistent feeling of sadness [1], affects approximately 280 million people globally [2]. It is one of the leading causes of the global burden of disease [3], thus posing a challenging public health issue. Many studies have documented robust relationships between depression and hopelessness and subsequent suicidal thoughts and behaviors [4]. Apart from its debilitating impact on the sufferer, depression also affects their close environment, as caregivers of individuals with depression often endure emotional and physical challenges, increasing the risk of experiencing psychological issues themselves [5]. Existing literature supports that there is a great variety of risk factors for depression. Sociodemographic factors, such as gender, marital status, age, educational level, and unemployment; daily habits, such as physical activity, diet, and sleep disturbances; and a wide variety of chronic physical diseases have been found to be related to depression with complex bi-directional relationships [6,7,8,9,10]. The pathogenesis of depression is associated with both genetic and environmental factors, with environmental features potentially having the greatest influence [11]. Due to the detrimental effects on people’s health, early diagnosis of depression is essential.

Machine learning (ML) is a powerful artificial intelligence (AI) tool used by researchers in the medical field to predict, calculate, and generate patterns for specific diagnoses. Over the past two decades, ML has been widely used to process statistical data to predict possible outcomes of complex biological systems [12]. The goal of ML is to detect underlying patterns within a sequence of observations by performing specific tasks to analyze data points collected by the physician’s team, ultimately producing predictions or even enabling early diagnoses. ML is a combination of algorithms exploring how computer systems can learn rules from multiple examples without explicit programming [13]. ML is gaining prominence in the field of medicine, demonstrating impressive results in predicting survival and prognosis among patients [14]. ML algorithms can handle and analyze large datasets more efficiently than traditional methods, allowing for the extraction of meaningful insights and physical laws that might otherwise be missed [15]. Neural networks are vital components of ML algorithms, which are modeled after the human brain. They function via pattern recognition, diagnosis, and prognosis in neurology. In a recent study, neural networks have been seen to achieve 87% accuracy, suggesting that such models can effectively assist neurologists in diagnosing and understanding multiple sclerosis (MS) [16].

ML has not only been used in psychiatry but also in a vast number of specialties, including surgery, nephrology, and genomic medicine. In surgery, it has been used to analyze the surgeon’s technical skill by detecting instrument motion, recognizing patterns in video recordings, tracking eye movements, and determining the cognitive function of the surgeon [17]. Another function for the use of ML is the benefit to chronic kidney disease (CKD). CKD is known to be a costly disease, and thus, with the help of ML, physicians can proceed to reduce the costs and provide more care to a greater patient population. In primary care settings, these algorithms can help address the issue by triggering early nephrology referral and improving outcomes in kidney disease patients [18]. Another example is the use of these programs in the field of genomic medicine, where the scope of ML can sift through complex genomic data to identify existing patterns associated with diseases such as cancer. Here, applying ML can help detect mutations in lesions or tumors. This integration allows for the identification of customized treatment recommendations, ultimately leading to enhanced patient outcomes [19].

Neurological disorders such as stroke, spinal cord injury, and Parkinson’s disease require accurate diagnosis and long-term neurorehabilitation, as they cause chronic disability. Diagnoses made by neuroimaging and physiological tools are important for accurately guiding the subsequent rehabilitation [20]. “Neuroscience and AI share a long history of collaboration”, as Macpherson et al. [21] claim; AI and ML algorithms are able to sort through vast amounts of complicated data, such as neuroimaging sets, while recognizing specific patterns, making them valuable for prognosis and guidance in treatment [21]. Therefore, “these newer technologies can offer better rehabilitation outcomes and patient care through more personalized treatments based on (such) data” [20].

With regard to mental health disorders, there is currently no available FDA-approved AI application. However, considering the chronicity and the significant burden of psychiatric disorders, there is a significant need for the utilization of AI and ML algorithms to assist, especially in identifying individuals at risk [22]. Mental health illnesses can pose a challenge in terms of diagnosis, as their disease patterns are interchangeable and complex. In this case, AI and ML could potentially address the challenge through their capacity to analyze extensive patient data, “including medical records, genetic information, and behavioral patterns” [23], thus enhancing diagnostic accuracy. Utilizing AI in the field of mental health also has the potential to establish diagnoses more objectively and detect early stages of disease where signs are frequently overlooked [24].

Utilization of AI and ML algorithms for depression provides meaningful insight into the disease, more effective drug regimens, and some predictive ability regarding patient outcomes [25]. Diagnosis of depression can be challenging, as it is highly heterogeneous, while it can also be underdiagnosed, as many individuals do not seek medical care due to the perceived stigma [26]. In the case of depression, prevention is of utmost importance, even more so than the diagnosis, as preventative actions significantly limit prevalence [27]. AI and ML algorithms have the capacity to possibly predict the development of depression by simply identifying certain environmental factors that put an individual at greater risk [28].

AI and ML, in the context of depression, could potentially be used to identify even minor signs, suggesting the presence of the disease based on behavioral and linguistic patterns. For instance, the patient’s vocal tone and pattern could point the algorithm towards a direction ranging from major depressive disorder to mild anxiety. Additionally, AI algorithms show promise in the ability to analyze specific brain areas, such as the amygdala, anterior cingulate cortex, and prefrontal cortex, that have been linked with anxiety and depression based on neuroimaging data [29]. Elnaggar et al. (2024) emphasize the pivotal role of electroencephalogram (EEG) analysis in advancing AI-driven approaches for depression diagnosis, demonstrating how EEG signals can be effectively leveraged to identify depression biomarkers [30]. In the realm of social media, Hasib et al. (2023) provide a comprehensive review of machine learning and deep learning techniques applied to social network data for depression detection, highlighting the efficacy of these methods in analyzing user-generated content to identify depressive symptoms [31]. Expanding beyond depression, Altomi et al. (2024) introduce a deep learning-based framework for diagnosing autism spectrum disorder (ASD) using facial images [32]. Their methodology employs attentional feature fusion with NasNetMobile and DeiT networks to capture intricate patterns and facial characteristics pertinent to ASD identification.

The aim of our study is to explore the association between depression and certain environmental factors, such as demographic characteristics, socioeconomics, general health, and habits, using four machine learning methods. Identifying which factors show a positive association and which are protective would allow for the creation of an algorithm that could predict and accurately diagnose depression, leading to earlier diagnosis and therefore prevention of worse outcomes, as well as adequate adaptation of therapy and treatment, thus limiting depression prevalence.

## 2. Materials and Methods

### 2.1. Study Sample and Research Design

The sample for this cross-sectional study included 1227 subjects, consisting of 657 women (53.5%) and 570 men (46.5%), with an average age of 49.94 ± 14.87 years (ranging from 19 to 76; median age 50 years). The sample selection, which took place between September 2016 and June 2022, was based on a two-stage stratified sampling scheme of adult people (18 years and older) residing in the culturally diverse region of Thrace, the northeastern prefecture of Greece, which includes a wide range of national, ethnolinguistic, and religious communities. The sampling procedure’s first stage involved dividing Thrace into two strata based on population size: urban (40% of the total population) and semi-urban or rural (60% of the total population). In the second step, participants were selected proportionally to the size of each stratum using a technique that randomly generated phone numbers using the area code. The participants consented to be interviewed in their home by field researchers and to complete the study questionnaires in a one-hour interview after the study’s purpose was explained to them. More details about the research design of this study are reported in Serdari et al. [33]. The overall response rate was 72.2%, which is fairly good for Greek standards (compared to 44.5% and 72% in the studies of Paparrigopoulos et al. [34] and Touloumi et al. [35], respectively). With 42.7% of the final sample coming from urban regions, 57.3% from rural areas, 65.8% from Greek Christians, 29.2% from Greek Muslims, and 5.1% from Greek expatriates, the sampling plan ensured that the sample was chosen at random and was representative of the overall population of Thrace. Due to their unique habits and daily routines, the study did not include those under 18, pregnant women, night shift workers, residents of institutions for chronic illnesses, residents of correctional facilities, and residents of retirement homes.

### 2.2. Ethics

All the procedures included in the study were carried out according to the ethics standards of the Democritus University Ethics Committee, which approved the realization of the study according to the standards of the Declaration of Helsinki (1964) and its subsequent amendments. Finally, all the participants in the study granted their consent.

### 2.3. Questionnaire Design—Covariates

After an extensive literature review, with the collaboration of a psychiatrist, we started creating a questionnaire in order to identify factors significantly associated with the prevalence of depression in the adult population. Finally, a structured questionnaire was developed, consisting of three distinct categories: formal sociodemographic characteristics, lifestyle and dietary habits, and health-related characteristics. In particular, the participants were requested to provide the following information: (a) formal sociodemographic characteristics (gender, age, place of residence, education level, presence of child <6 years old, marital, cultural, financial, and employment status); (b) lifestyle and dietary habits (smoking, alcohol consumption, daily consumption of coffee, adherence to choice of Mediterranean diet [36], physical activity, midday sleep, and duration of sleep); and (c) characteristics related to health (subjective general health status, body mass index [37], chronic disease morbidity, number of chronic diseases illnesses, anxiety [38], depression [39], family history of depression, traumatic events in the life of the participants, presence of insomnia or somnolence, and sleep quality) [40,41,42].

### 2.4. Assessment of Depression

The Greek version of the Beck Depression Inventory (BDI) [39] was used to evaluate depression symptoms. The BDI is a popular tool to measure typical depressive symptoms and behaviors. It comprises 21 self-reported Likert scale items, each of which is rated by respondents using a four-point scale ranging from 0 (i.e., I do not feel sad) to 3 (i.e., I am so sad and unhappy that I cannot stand it) based on how each item applied to them over the previous two weeks. The overall score is the sum of all items, with greater values representing higher degrees of depression. Due to its high sensitivity, a total score of 13 is utilized as a screening threshold for major depression [43]. The Greek version of the BDI demonstrates very good internal consistency, with alpha coefficients of 0.85 for individuals who have visited a public mental health center and 0.92 for a healthy population; very good test–retest reliability, with a correlation coefficient of r = 0.89 between the two measurements; and high validity, with correlation coefficients ranging from 0.66 to 0.80 with other depression and anxiety scales [44].

### 2.5. Problem Definition

**Figure 1:**
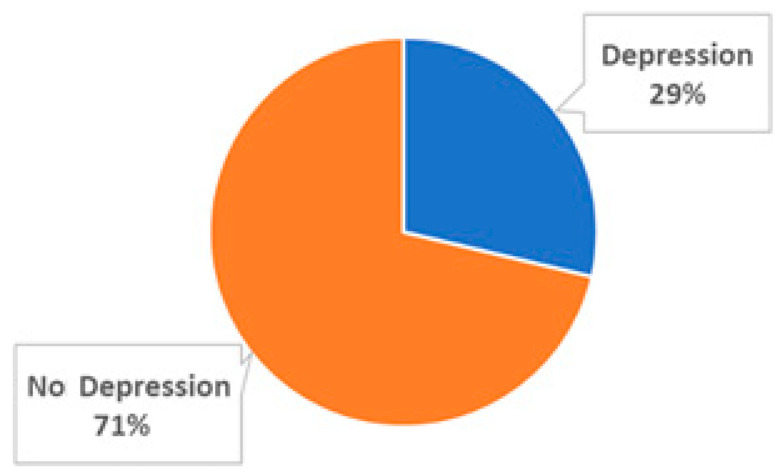
Grouping of the employed participants: No depression, Class 0 (*n* = 875 participants); Depression, Class 1 (*n* = 352 participants).

The participants were classified in a binary manner of “with depression” or “without depression”. Almost thirty percent of the entire cohort (29%; 352 participants; Class 1) presented with depression disorders, while the rest of them had no depression disorders (29%; 352 participants; Class 0). The employed dataset consists of 27 variables at baseline, with the target/dependent variable being the existence or non-existence of depression. Figure 1 presents the percentages of each class.

### 2.6. Machine Learning Workflow

**Figure 2:**
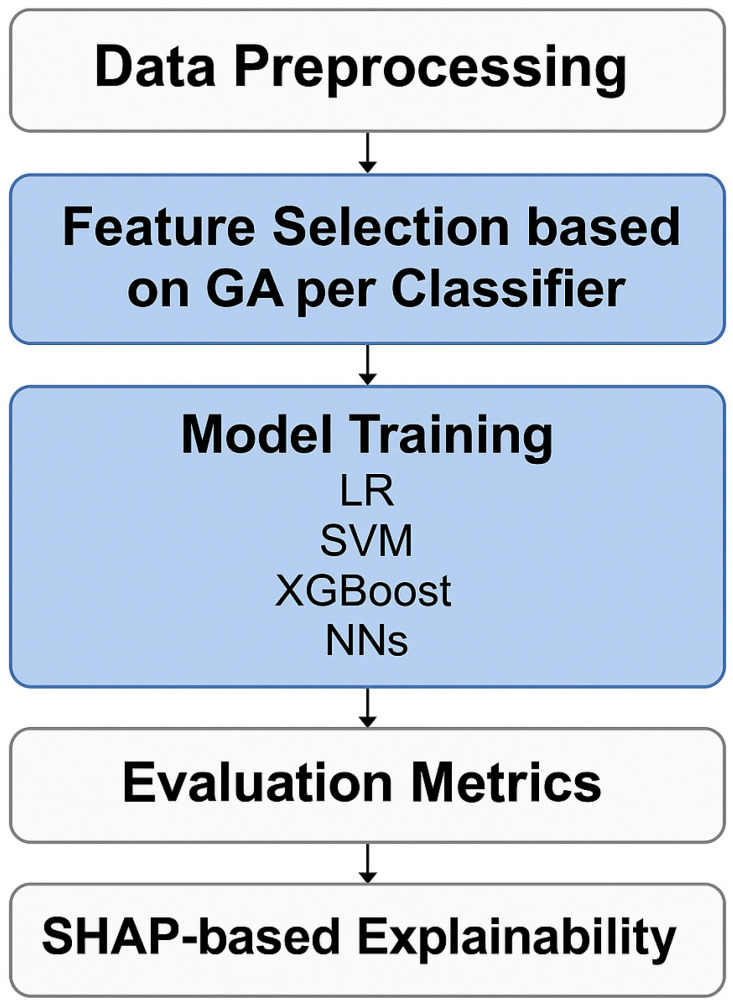
ML workflow.

To handle missing data in the dataset, the mode imputation strategy was used, which involves replacing missing values with the most frequently occurring value in the dataset. The study employed the genetic algorithm (GA) as a feature selection method to identify the optimal subset of features for improving the performance of the classifier. Four classifiers, namely, logistic regression (LR), support vector machines (SVMs), XGBoost, and neural networks (NNs), were used in the learning process, and a 70%/30% training/testing validation strategy was employed. These classifiers were selected based on their diverse strengths: LR serves as a simple and interpretable baseline; SVMs are effective in high-dimensional spaces and can model complex decision boundaries; XGBoost is powerful for capturing non-linear feature interactions and managing structured data; and NNs excel at recognizing intricate patterns in the data. Internal 10-fold cross-validation was used during the training phase to tune the hyperparameters after the undersampling step in the internal phase. The validation metrics included accuracy, recall, precision, F1 score, and specificity. The SHapley Additive exPlanations (SHAP) model assigns feature importance values using the concept of Shapley values from cooperative game theory and is a powerful tool for understanding the decision-making process of an ML model. All code for the development, training, and evaluation of the ML models was written in Python 3.9 utilizing the Scikit-learn library (https://scikit-learn.org/, accessed on 30 March 2025) as the primary framework for implementing ML algorithms and techniques. An ML pipeline was constructed to visually represent the methodology employed. The pipeline includes data preprocessing, FS using GA, training of four classifiers, evaluation through multiple metrics, and interpretation using SHAP values (Figure 2).

### 2.7. Statistical Analysis

Chi-squared analysis was used to evaluate whether the distribution of categorical variables, including subjects’ demographic characteristics, lifestyle habits, and health-related factors, differs significantly between individuals with depression and those without. The analysis revealed significant associations, indicating that variations in these factors are linked to differences in the prevalence of depression.

## 3. Results

In this section, the epidemiological profile and depression prevalence among subjects, the description of the 15 most significant risk factors, the testing results of the ML classifiers that were trained using the aforementioned risk factors, and the interpretation of the best ML model output are presented.

### 3.1. Epidemiological Profile and Depression Prevalence Among Subjects

The association of demographic characteristics with the prevalence of depression (Table 1) revealed that while gender was not significantly associated with depression (*p* = 0.145), age, marital status, cultural status, place of residence, education level, unemployment, and financial status showed significant differences in depression prevalence (all *p* < 0.001). In particular, older individuals, divorced subjects, those residing in rural areas, and participants with lower education or poorer financial conditions were more likely to experience depression. The absence of a child under six years old also showed a significant association (*p* = 0.029) with a higher prevalence of depression.

The association of lifestyle habits with the prevalence of depression (Table 2) revealed that depression was statistically significantly associated with alcohol consumption, coffee consumption, physical activity, and sleep duration (all *p* < 0.001). Subjects consuming more than four cups of coffee daily or those reporting short sleep duration had substantially higher depression rates, whereas higher levels of physical activity and lower or moderate alcohol consumption were linked to lower depression prevalence. In contrast, smoking status (*p* = 0.242), adherence to the Mediterranean diet (*p* = 0.080), and midday sleep (*p* = 0.101) did not show any statistically significant association with depression.

Health-related factors were strongly associated with the prevalence of depression (Table 3). Individuals with poor subjective health, chronic illnesses (especially those with multiple conditions), a positive family history of depression, exposure to traumatic life events, and anxiety symptoms were significantly more likely to be depressed (all *p* < 0.001). Additionally, the presence of insomnia (*p* = 0.042) and poor sleep quality (*p* = 0.008) was associated with higher depression rates, while BMI status (*p* = 0.103) and excessive daytime sleepiness (*p* = 0.704) did not demonstrate any statistically significant association with depression.

### 3.2. Feature Selection

Table 4 shows the 15 most significant risk factors with the highest level of significance identified using a genetic algorithm as a feature selection technique for predicting depression in a binary classification problem.

### 3.3. Testing Performance

Table 5 summarizes the testing performance metrics of a comparative analysis between the employed ML classifiers in this binary task. The XGBoost classifier achieved the best testing performance scores with the 15 most significant risk factors as they were selected from the GA algorithm. Specifically, 97.83% accuracy, 97.85% F1 score, 97.94% precision, 98.96% sensitivity, and 97.44% specificity were achieved by XGBoost. On the other hand, the lowest performance metrics were achieved by the LR classifier. In particular, LR achieved 79.95% accuracy, 79.04% F1 score, 78.82% precision, 79.95% sensitivity, and 90.84% specificity.

**Figure 3:**
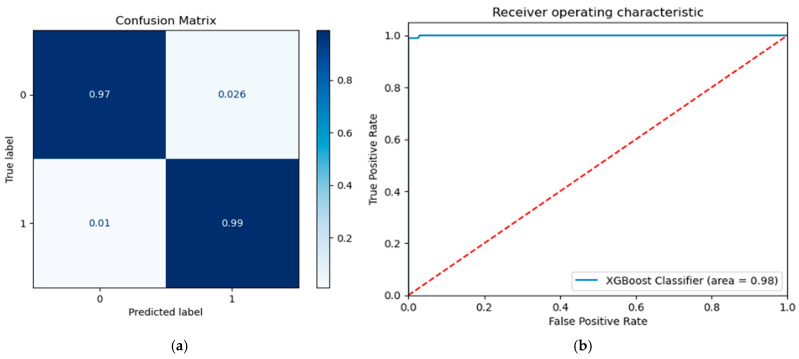
For the best ML classifier (XGBoost), (**a**) the confusion matrix and (**b**) receiver operating characteristics are presented.

Additionally, Figure 3 depicts the normalized confusion matrix and the receiver operating characteristics (0.98) for our best ML classifier. Specifically, the XGBoost classifier achieved 0.99 sensitivity and 0.97 specificity in this binary task.

### 3.4. Explainability

**Figure 4:**
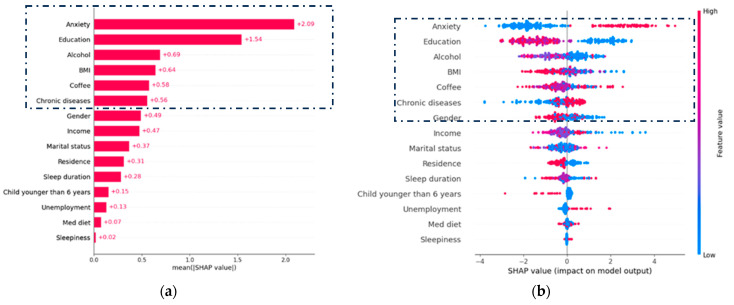
Risk factors on XGBoost ML classifier output for the diagnosis of depression. This figure presents (**a**) the SHAP feature importance and (**b**) the SHAP summary plot for the XGBoost trained on the risk factors selected by the GA. The dotted boxes in each image highlight the suggested features with the highest contribution.

In Figure 4, the effects of the 15 most significant risk factors on the output of the top-performing ML model (XGBoost) are illustrated. Figure 4a shows the mean absolute value of the SHAP values, which is an indicator of the SHAP global feature importance. Notably, the risk factors of anxiety, education, alcohol, BMI, and coffee had the greatest impact on the prediction output and were considered the most important features. Figure 4b displays the effect of each feature on the output of the final model (XGBoost) applied to the depression dataset. The features are sorted based on the sum of their SHAP value magnitudes across all samples. SHAP values are based on game theory and assign an importance value to each feature in a model. Features with positive SHAP values positively impact the prediction, while those with negative values have a negative impact. The magnitude is a measure of how strong the effect is.

The color of each feature represents its value (blue for low and red for high). This analysis reveals that high levels of anxiety among the participants lead to an increase in their predicted depression status. Moreover, high consumption of coffee, chronic diseases, unemployment, a Mediterranean diet, and sleepiness were associated with an increased risk of depression. On the contrary, higher education level, excessive drinking versus moderate drinking, higher BMI, being female, high income, residence in the country, and long sleep durations were associated with a reduced risk of depression.

## 4. Discussion

This study investigated the association between depression and multiple environmental factors, including sleep patterns, BMI, and diet. Data were collected through random phone number sampling, achieving a response rate of 72%. Participants completed a one-hour interview with healthcare professionals via phone call from their homes. The collected data were analyzed using multiple ML algorithms, including LR, NNs, SVMs, and XGBoost, with XGBoost demonstrating the highest reliability and accuracy. SHAP analysis identified several environmental factors with either positive or negative impacts on depression development. Although some SHAP-ranked features differ from those selected by GA, this reflects their differing objectives. GA identifies features enhancing classifier performance, while SHAP highlights those with the strongest influence on model output. In this discussion, we compare our findings to previous studies to better understand the factors influencing the prevalence and diagnosis of depression.

The prevalence of depression in the present study was high (28.7%), aligning with Kokaliari [45], who reported a 22.5% prevalence of moderate to severe depression within the Greek population. Similarly, Papadopoulos et al. [46] identified a high prevalence among individuals over 60 years of age living in rural Greece. Our study utilized the Greek version of the Beck Depression Inventory, which, while more effective as a screening tool than a diagnostic one, reliably identifies individuals at high risk or already experiencing depression [47].

Increased depression prevalence was observed among Greek Muslims (36.9%) and Greek expatriates (41.9%), compared to 24% among indigenous Greeks. This supports the hypothesis that culturally diverse communities are associated with a higher risk of depression, consistent with findings by Bailey et al. [48], who identified exclusion, lower socioeconomic status, and limited access to psychiatric care as key factors. Furthermore, belonging to such a group often reduces the likelihood of seeking mental health support [48], despite evidence that any form of social identity can confer protection against mental illness [49].

Higher income and financial stability were associated with a decreased risk of depression; however, consistent with previous studies, a U-shaped relationship was observed. Depression was more prevalent at very low and very high-income levels, while mid- to high-income levels were protective [50,51]. These findings echo those of Stylianidis and Souliotis [52], who reported a significant impact of unemployment and financial hardship on depression and suicidality during the Greek economic crisis.

Among all factors, educational attainment emerged as the strongest protective predictor against depression, supporting the findings of Biswas et al. [53]. Nevertheless, when coupled with unemployment, particularly during adolescence, the protective effect of education diminished. Unemployed adolescents with higher education levels showed increased anxiety and depression symptoms, driven by societal and familial pressures. Thus, the interplay between education and other socioeconomic factors should be considered when evaluating depression risk. Including vocational and skills-based courses in curricula could enhance future employment prospects [53].

Anxiety was the most significant risk factor for depression in our study, in line with existing research showing that approximately 85% of depression cases are comorbid with anxiety disorders [54,55,56]. Generalized anxiety disorder, in particular, frequently precedes depression [57]. Avoidant behaviors driven by anxiety can evolve into depression [58]. Treatments such as cognitive behavioral therapy (CBT) and antidepressants benefit both conditions [56], and neuroimaging studies suggest shared brain alterations in emotion-processing circuits [59]. The STAR*D study further highlighted that comorbid anxiety-depression leads to more severe depressive episodes and increased suicide risk [60].

Interestingly, our findings diverged from the widely reported trend of higher depression rates among females, as we found a lower prevalence among women. Although epidemiological studies commonly show a 2:1 female-to-male ratio for major depression [61], differences in symptom presentation—internalizing symptoms in men versus externalizing in women [62]—and sensitivity to interpersonal versus extrinsic factors [63] could explain this discrepancy in our sample.

Contrary to expectations, heavy drinking was negatively associated with depression risk. Depression prevalence decreased with higher alcohol consumption and increased among moderate or non-drinkers. Although alcohol dependence has been linked to depression [64], some studies suggest moderate drinking may improve mood and cognitive function [65]. This complexity highlights the need for more nuanced evaluations.

Similarly, a higher BMI was negatively associated with depression risk in our study, whereas prior research, such as that by Kraus et al. [66], linked obesity with treatment-resistant depression and worse clinical outcomes. Badillo et al. [67] found obesity to be especially detrimental for men, largely mediated by poor sleep quality. Our findings align more closely with Cui et al. [68], who described a U-shaped relationship between BMI and mental health, suggesting that maintaining a healthy weight offers the best protection.

In terms of sleep, our findings revealed that both short and prolonged sleep durations were associated with depression, reflecting Zhai et al.’s meta-analysis [69]. Although some previous studies did not find a link between longer sleep duration and depression [70,71], our data, consistent with Badillo et al. [67], suggest that sleep disturbances, potentially driven by inflammation, biochemical, or genetic mechanisms, play a key role in depression development.

Caffeine consumption also emerged as a risk factor for depression, likely through its negative effects on sleep and anxiety. However, Narita et al. [72] found that black coffee, without additives, might have protective effects due to lower inflammation and maintained brain-derived neurotrophic factor (BDNF) levels. While moderate coffee intake has been linked to reduced depression risk in prior studies, our SHAP analysis suggests that high consumption (>4 cups/day) is positively associated with depression, possibly due to sleep disruption and increased anxiety. This divergence may reflect differences in population characteristics or confounding factors.

As expected, depression was more common among individuals with chronic diseases such as diabetes, arthritis, and asthma [73], consistent with Herrera et al. [74]. However, effective self-regulation and disease management appeared to mitigate the psychological burden for some patients.

In contrast to most studies [75,76,77], adherence to a Mediterranean diet (MD) was unexpectedly associated with a higher depression risk. Although traditionally protective, issues with low adherence or misreporting might explain this contradiction, as noted by Radkhah et al. [78]. Sánchez-Villegas et al. [75] demonstrated that while B vitamins showed a protective effect, omega-3 fatty acids did not have a significant impact.

Living in rural areas was generally protective against depression, consistent with findings by Pérès et al. [79], who cited stronger social support during the COVID-19 lockdown. However, Nam et al. [80] identified farmworkers as an exception due to unique occupational stressors.

In terms of model performance, XGBoost and NNs outperformed other ML models for predicting depression-associated factors. These findings align with those of Qasrawi et al. [81], who suggested that ML models can help healthcare professionals implement preventive interventions. XGBoost was particularly noted for its superior modeling capabilities over LR, SVM, and decision trees, as supported by Sharma and Verbeke [82] and Kessler et al. [83]. The consistent advantage of ML methods underlines the importance of using sophisticated algorithms, especially as the number of predictive factors increases. However, challenges remain. Richter et al. [84] noted inconsistencies in ML performance across different datasets and methods, suggesting a need for greater standardization.

Specifically, we selected XGBoost as one of the classifiers due to its demonstrated effectiveness in handling structured, tabular data and its capacity to model complex, non-linear relationships between features. Compared to traditional models like LR and SVMs, XGBoost offers enhanced performance by employing an ensemble of decision trees optimized through gradient boosting techniques. This allows it to capture intricate patterns in the data that linear models might overlook. Furthermore, XGBoost incorporates regularization parameters to prevent overfitting, making it robust across various datasets. While NNs are powerful in modeling non-linear relationships, they often require larger datasets and more computational resources. XGBoost, on the other hand, achieves a balance between performance and computational efficiency, making it particularly suitable for our dataset and research objectives.

## 5. Limitations

Despite the valuable insights gained from this study, several limitations must be acknowledged. First, the cross-sectional design prevents the establishment of causal relationships between environmental factors and depression. Second, self-reported data collected via phone interviews may introduce recall bias or social desirability bias, potentially affecting the accuracy of responses. Third, although random sampling was employed, selection bias cannot be fully excluded, particularly given the 28% non-response rate. Additionally, while the Greek Beck Depression Inventory is a validated screening tool, it is not a definitive diagnostic instrument, which may influence the estimated prevalence rates. Finally, although ML models such as XGBoost and NNs demonstrated strong predictive ability, model performance could vary with different datasets or demographic contexts, and external validation with independent samples is necessary to confirm generalizability.

## 6. Future Directions

Most predictive studies for depression to date have relied on relatively small sample sizes, especially in the context of treatment response prediction. While small datasets are valuable for model development and hypothesis generation, larger and more diverse cohorts are crucial for building robust and generalizable machine learning models. As such datasets become available, applying more rigorous validation strategies, such as higher k-fold cross-validation or external validation using independent datasets, will be critical to ensure model reproducibility and clinical applicability. In parallel, integrating multimodal data sources such as neuroimaging, genetic profiles, and electronic health records could enhance model performance by capturing the complex, multifactorial nature of depression. Feature selection techniques and algorithm choices should also be adapted to handle high-dimensional, heterogeneous data effectively. Ultimately, future research should focus on translating predictive models into clinically deployable tools, enabling personalized treatment strategies and improving outcomes in real-world psychiatric care.

## 7. Conclusions

In summary, depression is a pathological illness that can affect individuals of any age and gender. It is also more frequently observed in individuals with comorbid physical illnesses. ML approaches have shown significant promise in aiding the diagnosis of various mental health conditions, including schizophrenia, depression, bipolar disorder, autism spectrum disorders, and post-traumatic stress disorder. To detect such conditions, data derived from patients’ social profiles, general clinical health status, and sensory mobile applications can be analyzed. In the present study, we examined contemporary research on the diagnosis of depression using ML-based approaches. Our aim was to provide information on the fundamental concepts of ML algorithms employed in mental health, particularly depression, and to explore their practical application. The results indicate that XGBoost outperforms traditional projection methods, demonstrating superior adaptability in predicting depression. Importantly, XGBoost’s benefits extend beyond diagnosis, offering potential for predicting the future development of the disorder. A key advantage of this method is its applicability to individualized analysis. SHAP analysis identified anxiety, education level, alcohol consumption, and BMI as the most influential predictors of depression. These findings emphasize the value of explainable ML tools like SHAP in improving transparency and guiding targeted interventions.

Future studies could focus on expanding the dataset size to enhance training and validation processes, thereby improving the model’s performance and reliability for clinical applications. As depression is a leading cause of impaired quality of life and remains challenging to predict, the application of advanced ML models like XGBoost offers a promising new direction in the therapeutic management of the disorder. The identified risk factors could contribute to the development of intelligent mental healthcare systems capable of detecting early signs of depressive symptoms, including within workplace environments.

## Figures and Tables

**Table 1:** Prevalence of depression in relation to subjects’ demographic characteristics.

		Depression		
	Number (%)	Frequency	Proportion (%)	*p* Value
Gender				0.145
Males	570 (46.5)	152	26.7	
Females	657 (53.5)	200	30.4	
Age (years)				<0.001
≤40	341 (27.8)	42	12.3	
41–60	571 (46.5)	164	28.7	
>60	315 (25.7)	146	46.3	
Marital status				<0.001
Married	825 (67.2)	257	31.2	
Single	252 (20.5)	41	16.3	
Divorced	102 (8.3)	42	41.2	
Widowed	48 (3.9)	12	25.0	
Cultural status				<0.001
Greek Christians	807 (65.7)	194	24.0	
Greek Muslims	358 (29.2)	132	36.9	
Expatriated Greeks	62 (5.1)	26	41.9	
Place of residence				<0.001
Urban	524 (42.7)	88	16.8	
Rural	703 (57.3)	264	37.6	
Education level				<0.001
Low	406 (33.1)	211	52.0	
Medium	431 (35.1)	98	22.7	
High	390 (31.8)	43	11.0	
Presence of child <6 years				0.029
No	1128 (91.9)	333	29.5	
Yes	99 (8.1)	19	19.2	
Unemployment				<0.001
No	1121 (91.4)	303	27.0	
Yes	106 (8.6)	49	46.2	
Financial status				<0.001
Low	614 (50.0)	213	34.7	
Medium	258 (21.0)	33	12.8	
High	180 (14.7)	29	16.1	

**Table 2:** Prevalence of depression in relation to subjects’ lifestyle habits.

		Depression		
	Number (%)	Frequency	Proportion (%)	*p* Value
Smoking status				0.242
Never/ex-smoker	808 (65.9)	223	27.6	
Current smoker	419 (34.1)	129	30.8	
Alcohol consumption				<0.001
None	621 (50.6)	212	34.1	
1–3 glasses/week	316 (25.8)	69	21.8	
4–6 glasses/week	215 (17.5)	42	19.5	
>6 glasses/week	75 (6.1)	29	38.7	
Coffee consumption				<0.001
None	113 (9.2)	33	29.2	
1–2 cups/day	723 (58.9)	179	24.8	
3–4 cups/day	322 (26.2)	99	30.7	
>4 cups/day	69 (5.6)	41	59.4	
Adherence to Mediterranean diet				0.080
Low	968 (78.9)	289	29.9	
High	259 (21.1)	63	24.3	
Physical activity				<0.001
Low	1031 (84.0)	321	31.1	
High	196 (16.0)	31	15.8	
Midday sleep				0.101
No	520 (42.4)	162	31.2	
Yes	707 (57.6)	190	26.9	
Sleep duration				<0.001
Short	273 (22.2)	130	47.6	
Normal	780 (63.6)	176	22.6	
Long	174 (14.2)	46	26.4	

**Table 3:** Prevalence of depression in relation to subjects’ health-related characteristics.

		Depression		
	Number (%)	Frequency	Proportion (%)	*p* Value
BMI status				0.103
Normal	415 (33.8)	113	27.2	
Overweight	352 (28.7)	91	25.9	
Obese	460 (37.5)	148	32.2	
Subjective health status				<0.001
Good	941 (76.7)	168	17.9	
Bad	286 (23.3)	184	64.3	
Morbidity of chronic illness				<0.001
No	534 (43.5)	94	17.6	
Yes	693 (56.5)	258	37.2	
Number of chronic diseases				<0.001
None	534 (43.5)	94	17.6	
One	360 (29.3)	97	26.9	
Two	208 (17.0)	87	41.8	
More than two	125 (10.2)	74	59.2	
Family history of depression				<0.001
No	812 (66.2)	199	24.5	
Yes	415 (33.8)	153	36.9	
Traumatic events in life				<0.001
No	716 (58.4)	155	21.6	
Yes	511 (41.6)	197	38.6	
Anxiety symptoms				<0.001
No	813 (66.3)	119	14.6	
Yes	414 (33.7)	233	56.3	
Excessive daytime sleepiness				0.704
No	1120 (91.3)	323	28.8	
Yes	107 (8.7)	29	27.1	
Presence of insomnia				0.042
No	1015 (82.7)	279	27.5	
Yes	212 (17.3)	73	34.4	
Sleep quality				0.008
Good	765 (62.3)	199	26.0	
Bad	462 (37.7)	153	33.1	

**Table 4:** Ranking of the most informative risk factors in depression diagnosis using a genetic algorithm.

Risk Factor	Description	Type of Variable
Gender	Gender (male/female)	Categorical
Marital status	Marital status (single/married/divorced/widowed)	Categorical
Residence	Area of residence (urban/rural)	Categorical
Education	Education level (low/medium/high)	Categorical
Unemployment	Unemployment (no/yes)	Categorical
Income	Income (low/medium/high)	Categorical
Chronic diseases	Chronic diseases (no/yes)	Categorical
BMI	Body mass index (normal/overweight/obese)	Categorical
Alcohol	Alcohol consumption/week (none/1–3 glasses/4–6 glasses/>6 glasses)	Categorical
Coffee	Coffee consumption/day (none/1–2 glasses/3–4 glasses/>4 glasses)	Categorical
Mediterranean diet	Adherence to Mediterranean diet (no/yes)	Categorical
Child <6 years	Presence of a child younger than 6 years of age (no/yes)	Categorical
Sleep duration	Sleep duration (short/normal/long)	Categorical
Sleepiness	Excessive daytime sleepiness (no/yes)	Categorical
Anxiety	Anxiety (no/yes)	Categorical

**Table 5:** Metrics of testing performance for the employed classifiers.

Classifier	Accuracy (%)	F1 Score (%)	Precision (%)	Sensitivity (Recall) (%)	Specificity (%)	Hyperparameters
**LR**	79.95	79.04	78.82	79.95	90.48	C: 1, penalty: l2
**SVM**	95.66	95.64	95.63	95.66	97.80	C: 10, kernel: rbf
**XGBoost**	**97.83**	**97.85**	**97.94**	**98.96**	**97.44**	**gamma: 0, max_depth: 7, min_child_weight: 1**
**NN**	97.02	97.03	97.06	97.02	97.44	activation: tanh, alpha: 0.0001, hidden_layer_sizes: (10, 20, 50), learning_rate: constant, solver: adam

## Data Availability

All data are available upon request.
